# P58^IPK^: A Novel “CIHD” Member of the Host Innate Defense Response against Pathogenic Virus Infection

**DOI:** 10.1371/journal.ppat.1000438

**Published:** 2009-05-22

**Authors:** Alan G. Goodman, Jamie L. Fornek, Guruprasad R. Medigeshi, Lucy A. Perrone, Xinxia Peng, Matthew D. Dyer, Sean C. Proll, Sue E. Knoblaugh, Victoria S. Carter, Marcus J. Korth, Jay A. Nelson, Terrence M. Tumpey, Michael G. Katze

**Affiliations:** 1 Department of Microbiology, University of Washington, Seattle, Washington, United States of America; 2 Graduate Program in Bioengineering, University of Washington, Seattle, Washington, United States of America; 3 Vaccine and Gene Therapy Institute, Oregon Health and Science University, Beaverton, Oregon, United States of America; 4 Influenza Division, National Center for Immunization and Respiratory Diseases, Centers for Disease Control and Prevention, Atlanta, Georgia, United States of America; 5 Animal Health Shared Resources, Fred Hutchinson Cancer Research Center, Seattle, Washington, United States of America; 6 Washington National Primate Research Center, Seattle, Washington, United States of America; University of California San Francisco, United States of America

## Abstract

To support their replication, viruses take advantage of numerous cellular factors and processes. Recent large-scale screens have identified hundreds of such factors, yet little is known about how viruses exploit any of these. Influenza virus infection post-translationally activates P58^IPK^, a cellular inhibitor of the interferon-induced, dsRNA-activated eIF2α kinase, PKR. Here, we report that infection of P58^IPK^ knockout mice with influenza virus resulted in increased lung pathology, immune cell apoptosis, PKR activation, and mortality. Analysis of lung transcriptional profiles, including those induced by the reconstructed 1918 pandemic virus, revealed increased expression of genes associated with the cell death, immune, and inflammatory responses. These experiments represent the first use of a mammalian infection model to demonstrate the role of P58^IPK^ in the antiviral response. Our results suggest that P58^IPK^ represents a new class of molecule, a cellular inhibitor of the host defense (CIHD), as P58^IPK^ is activated during virus infection to inhibit virus-induced apoptosis and inflammation to prolong host survival, even while prolonging viral replication.

## Author Summary

Respiratory illness caused by influenza virus is the seventh leading cause of death in the United States, and there is considerable interest in determining how the virus and host interact during infection and how the virus causes disease. Because influenza virus encodes only eight genes, it is dependent upon cellular proteins and processes for the generation of new viruses. One of these proteins is P58^IPK^, the activation of which has long been thought to be required for efficient viral replication. To study how P58^IPK^ affects the ability of influenza virus to replicate and cause disease, we infected a strain of mice that lacks the gene for P58^IPK^. We found that viruses replicated just as efficiently in mice lacking the P58^IPK^ gene, but that these mice were more susceptible to a fatal infection. The increased mortality rate in these mice was due to greater lung pathology and a heightened inflammatory response. Our results show that P58^IPK^ represents a new type of protein that benefits the host by reducing pathology and prolonging survival, even though it may also benefit the virus by allowing it to replicate for a longer period of time.

## Introduction

Through a series of signaling mechanisms, the mammalian innate immune system recognizes and responds to pathogens to protect the host during infection. The response is initiated when pathogen-associated molecular patterns (PAMPs), present in microbial proteins or RNAs, engage cellular pathogen-recognition receptors (PRRs) such as RIG-I, MDA5, or the toll-like receptors [1]. Upon engagement, these PRRs activate proteins such as IRF3, IRF7, and NF-κB, which translocate to the nucleus and induce the type I interferon (IFN) response [2]. Type I IFNs in turn induce the expression of numerous IFN-stimulated genes (ISGs), including IFNγ, interleukin (IL)-6, and the dsRNA-activated protein kinase, PKR [3]. Downstream targets of PKR include the α subunit of eukaryotic initiation factor 2 (eIF2α), NF-κB, and additional ISGs [4]–[6], thus amplifying the IFN response via a positive feedback mechanism.

P58^IPK^ is a cellular inhibitor of PKR that is activated at the post-translational level in response to influenza virus infection [7]. The activation of P58^IPK^ results in reduced levels of PKR-mediated eIF2α phosphorylation, which has long been thought to benefit influenza virus by maintaining a high rate of viral protein translation [8],[9]. P58^IPK^ is also activated at the transcriptional level in response to endoplasmic reticulum (ER) stress. During ER stress, P58^IPK^ inhibits another eIF2α kinase, PERK, which functions to regulate protein synthesis during the unfolded protein response [10],[11]. P58^IPK^ also plays a larger role in the protein processing efficiency of the ER by binding to misfolded proteins and acting as a co-chaperone [12]–[14].

Recently, we showed that influenza virus infection of mouse embryonic fibroblasts (MEFs) lacking P58^IPK^ results in increased eIF2α phosphorylation and decreased viral mRNA translation [8]. This effect was due to P58^IPK^ functioning through a PKR-dependent mechanism that is independent of PERK. We also showed that P58^IPK^ functions similarly during infection with vesicular stomatitis virus (VSV) or reovirus. These findings again suggested that influenza virus benefits from P58^IPK^ activation, presumably to the detriment of the host.

Here, we sought to determine for the first time the role of P58^IPK^ using an *in vivo* virus infection model. To this end, we infected mice lacking P58^IPK^ with either the mouse-adapted A/PR/8/34 (PR8) strain or the reconstructed 1918 (r1918) pandemic influenza virus. We then examined viral replication, lung pathology, PKR and eIF2α phosphorylation, cytokine expression, and global gene transcriptional profiles. We found that influenza virus infections were more lethal in mice lacking P58^IPK^, due to increased lung pathology and inflammation. Therefore, just as P58^IPK^ plays a role in restoring homeostasis during ER stress through its interaction with PERK, P58^IPK^ may function during virus infection to restore homeostasis during the antiviral and inflammatory response through its interaction with PKR. Our findings suggest that P58^IPK^ represents a new class of molecule that is activated during virus infection to help regulate the host antiviral response. While a number of host proteins exist which are crucial in determining the antiviral response, P58^IPK^ is the first to be identified in a mammalian system which inhibits the defense response for proper development of the antiviral state. We coin this new class of regulators “Cellular Inhibitors of the Host Defense” (CIHDs).

## Results

### P58^IPK−/−^ mice exhibit a higher influenza virus-induced mortality rate

**Figure 1:**
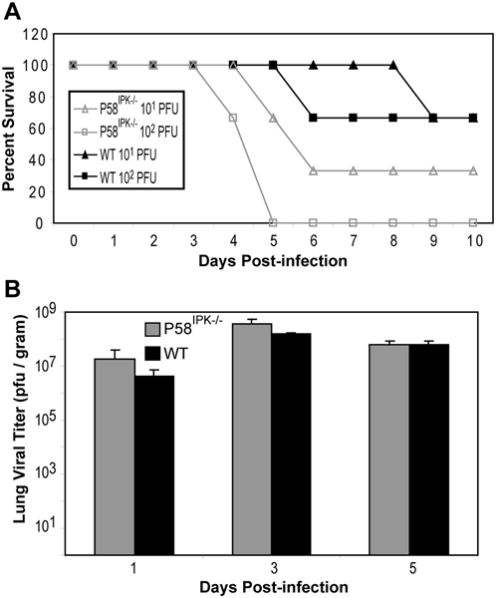
P58^IPK−/−^ mice exhibit increased mortality to influenza virus infection yet similar levels of virus replication. (A) Three P58^IPK−/−^ and wild-type mice were infected with 10 or 100 PFU of the PR8 strain of influenza virus. The number of surviving mice, as determined by having at least 20% of starting weight, at each day is shown. (B) Three P58^IPK−/−^ and wild-type mice infected with 10^3^ PFU of PR8 were sacrificed at 1, 3, or 5 days post infection. Levels of infectious virions in diaphragmatic lung homogenates were determined by triplicate plaque assay on MDCK cells. The results represent the mean activity of 3 independent samples±standard deviation.

To determine the role of P58^IPK^ using a *bona fide* mammalian infection model, we infected P58^IPK−/−^ and wild-type mice with a series of doses (10^1^ to 10^5^ PFU) of the PR8 strain of influenza virus. Although P58^IPK−/−^ and wild-type mice exhibited similar weight loss (morbidity) over time (data not shown), mortality was markedly increased in P58^IPK−/−^ mice. This phenotype was particularly pronounced at lower infectious doses (Figure 1A). When infected with 10^2^ PFU, all P58^IPK−/−^ mice died by day 5, whereas only 33% of wild-type mice died by day 6, and the others recovered. This corresponded to a fifty percent lethal dose (LD_50_) of 10^1.3^ PFU for P58^IPK−/−^ mice and 10^2.0^ PFU for wild-type mice. The increased mortality in P58^IPK−/−^ mice was not due to increased viral load, since viral titers in the lungs of all infected mice were similar at each time point (Figure 1B). Furthermore, mortality was not caused by viral attachment or initial infection alone, since experiments with a replication-deficient virus (PR8ΔNS1) [15] did not produce disease in either mouse genotype (data not shown).

**Figure 2:**
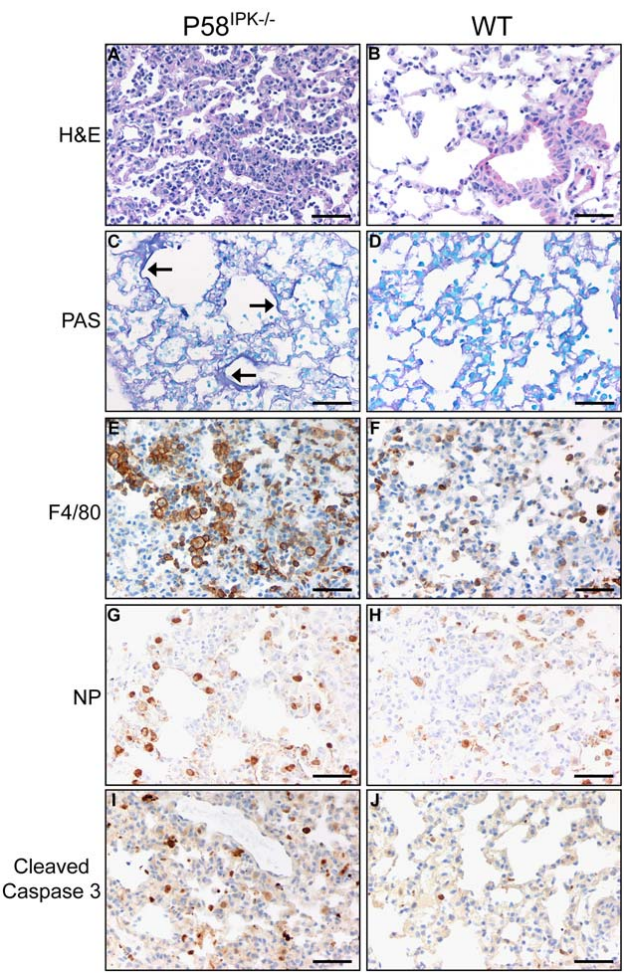
P58^IPK−/−^ mouse lungs exhibit increased pathology during influenza virus infection. P58^IPK−/−^ and wild-type (WT) mice were infected with 10^3^ (A,B, E–H), 10^4^ (I,J), or 10^5^ (C,D) PFU of the PR8 strain of influenza virus and stained for hematoxalin and eosin (H&E) (A,B), periodic acid-Schiff (PAS) (C,D), F4/80 (E,F), influenza virus NP (G,H), or cleaved caspase 3 (I,J) at 5 days post infection. This shows that, while both P58^IPK−/−^ and wild-type mouse lungs contain similar amounts of infected cells, P58^IPK−/−^ mice exhibit increased alveolitis and peribrochiolitis, marked by macrophage and neutrophil infiltration, hyaline membrane formation, and caspase activation. Arrows denote hyaline membranes. Bar = 50 µm.

While both P58^IPK−/−^ and wild-type mice exhibited great lung pathology when infected with high doses of PR8, microscopic examination revealed increased lung pathology in P58^IPK−/−^ mice, as compared to wild-type mice, infected with a lower dose of PR8. This was marked by moderate to severe alveolitis and peribronchiolitis, macrophage and neutrophil infiltration, and associated hemorrhage and edema (Figure 2A). By contrast, wild-type mice had mild alveolitis and peribronchiolitis with lower levels of neutrophils, where infiltration of inflammatory cells is scant (Figure 2B). Alveolitis was observed as early as day 1 post infection in P58^IPK−/−^ mice and inflammation increased in severity until day 5 (Figure S1). Other histological features which were more pronounced in P58^IPK−/−^ mice were multifocal intraluminal inflammation with necrotic debris and the formation of hyaline membranes adjacent to alveolar walls (Figure 2C–D). Staining for the macrophage marker F4/80 revealed the presence of greatly increased numbers of macrophages in the lungs of infected P58^IPK−/−^ mice as compared with wild-type mice (Figure 2E–F). The presence of macrophages was observed by day 1 post infection in P58^IPK−/−^ mice, whereas their presence was not detected until day 3 post infection in wild-type mice (Figure S2).

Even though there was increased pathology in the lungs of infected P58^IPK−/−^ mice, a similar number of cells stained positive for influenza virus NP in both P58^IPK−/−^ and wild-type mice (Figure 2G–H) throughout the course of infection (Figure S3). Thus, differences in pathology did not appear to be due to differences in the amount of virus present in lung cells.

Influenza virus infection can induce apoptosis [16], and more specifically, the caspase cascade can be activated through the expression of IFN [17]. Therefore, to determine if apoptosis was induced in the lungs of P58^IPK−/−^ mice, we looked for the presence of cleaved caspase 3. Caspase 3 is downstream of cleaved caspase 8, and it cleaves other caspases as well as poly(ADP) ribose polymerase [18]. This analysis revealed increased staining of cleaved caspase 3 in the lungs of P58^IPK−/−^ mice compared with that detected in wild-type mice (Figure 2I–J), but no cleaved caspase 3 was observed in the lungs of mock-infected animals (Figure S4). Increased apoptosis in the lungs of P58^IPK−/−^ mice is consistent with our previous findings that P58^IPK^ has anti-apoptotic properties [19]. Our observation of increased apoptosis and mortality in mice lacking P58^IPK^ is also consistent with previous reports of increased apoptosis being a marker for fatal influenza virus infection [20].

### P58^IPK−/−^ mice exhibit increased levels of eIF2α and PKR phosphorylation in response to infection

**Figure 3:**
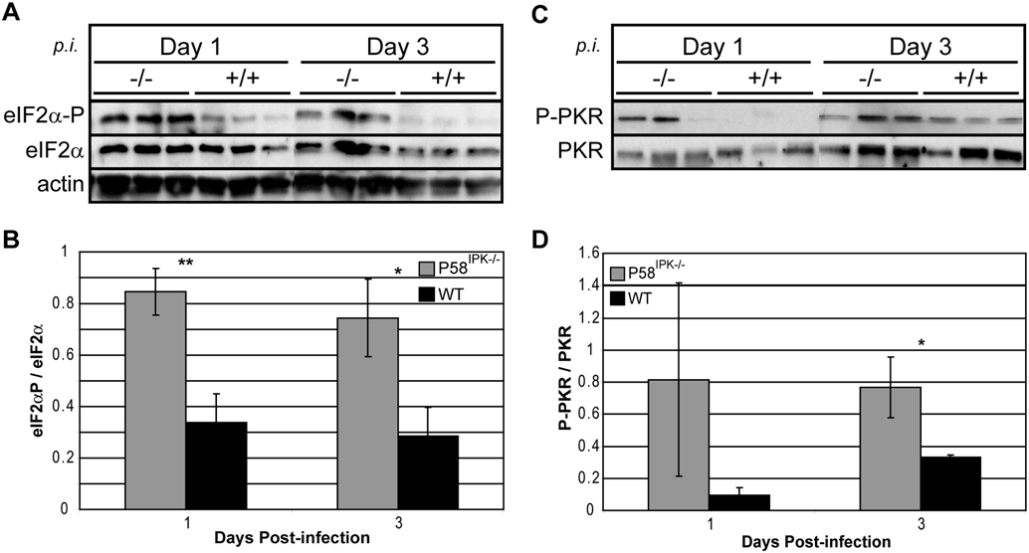
P58^IPK−/−^ mouse lungs exhibit increased eIF2α and PKR phosphorylation during influenza virus infection. Three P58^IPK−/−^ and wild-type mice were infected with 10^4^ (A,B) or 10^3^ (C,D) PFU of the PR8 strain of influenza virus. At 1 and 3 days post infection, azygous and apical lung lobes were excised and homogenized. (A) Equivalent concentrations of protein in lung homogenates were subjected to SDS-10% PAGE. The levels of phosphorylated or total eIF2α and actin were determined by immunoblot analysis. (B) Densitometry analysis of (A). The density of the eIF2α-P band was normalized to the density of the total eIF2α band, which is represented graphically. (C) 400 µg of total protein from lung homogenates was used for immunoprecipitation with an antibody for total PKR. Following immunoprecipitation, protein samples were clarified by SDS-10% PAGE and the levels of phosphorylated or total PKR were determined by immunoblot analysis. (D) Densitometry analysis of (C). The density of the P-PKR band was normalized to the density of the total PKR band, which is represented graphically. The results represent the mean activity of 3 biologically independent samples±standard deviation. *P*-values from a two-tailed *t*-test assuming non-equal variance are indicated (*: *P*<0.05, **: *P*<0.05).

Influenza virus infection of MEFs devoid of P58^IPK^ results in increased PKR activation and eIF2α phosphorylation relative to wild-type MEFs [8]. In the present study, we determined the amount of eIF2α and PKR phosphorylation in the lungs of P58^IPK−/−^ and wild-type mice infected with PR8. We observed increased eIF2α phosphorylation in the lungs of influenza virus-infected P58^IPK−/−^ mice throughout infection (Figure 3A). Quantification of the results showed that eIF2α phosphorylation was significantly increased in P58^IPK−/−^ mouse lungs at days 1 and 3 post infection (Figure 3B). Increased and prolonged eIF2α phosphorylation is reported to lead to apoptosis [21], which is consistent with our findings of increased levels of caspase 3 activation and mortality in mice lacking P58^IPK^. Further investigation revealed that there was increased PKR phoshorylation (Figure 3C–D), but not PERK phosphorylation (data not shown), in the absence of P58^IPK^, resulting in increased eIF2α phosphorylation.

### P58^IPK−/−^ mice exhibit increased levels of inflammatory and immune response genes early after infection

Our traditional virology, histology, and biochemical approaches indicated that influenza virus infection of P58^IPK−/−^ mice was associated with increased lung pathology characterized by macrophage infiltration, apoptosis, and increased levels of eIF2α phosphorylation. Each of these effects have been associated with severe influenza virus infection [20],[22],[23]. In order to discover potentially new mechanisms that might contribute to increased mortality in mice lacking P58^IPK^, we used oligonucleotide microarrays to profile the host transcriptional response to infection.

For our microarray analyses, P58^IPK−/−^ and wild-type mice were infected intranasally with 10^3^ PFU of PR8, or mock-infected with PBS alone, and sacrificed at days 1, 3, and 5 post infection. RNA was then isolated from left lung lobes for gene expression profiling. Analyses were performed by comparing RNA isolated from each individual animal against a pool of RNA from genotype-matched mock-infected animals. To perform a direct comparison of gene expression profiles of P58^IPK−/−^ and wild-type mice, we used the re-ratio tool in Rosetta Resolver. This tool creates new ratio experiments from two or more existing ratio experiments that share a common reference, therefore removing the reference and focusing on the difference between two conditions. Next, working with one set of gene expression data at each time point, we used Gene Set Enrichment Analysis (GSEA) [24] to identify sets of genes that were differentially expressed between the P58^IPK−/−^ and wild-type mice.

GSEA determines how two distinct phenotypes differ in their gene expression profiles by ranking the significance of the gene ontology (GO) sets identified. Given a defined set of genes annotated with a certain GO function, GSEA determines whether the members of that set are randomly distributed throughout the ranked list or if they are found primarily at the top or bottom of that list. Those sets at the top or bottom of the list describe the phenotypic distinction between the two sets [24]. While traditional GO analysis uses a preselected set of genes created using an arbitrary fold-change or *P* value cutoff, GSEA examines the entire gene set [25].

**Figure 4:**
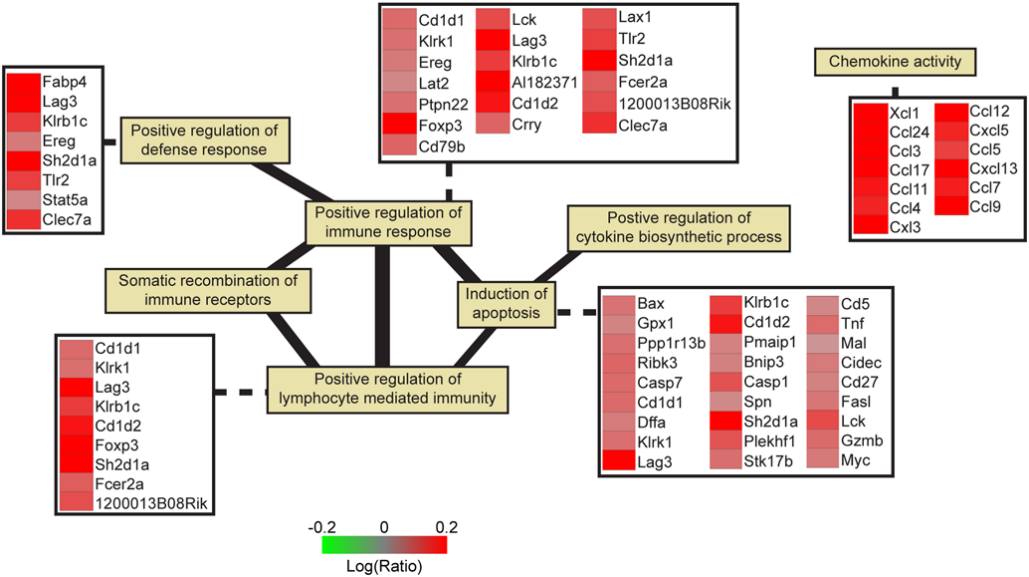
Inflammatory response genes are more up-regulated in influenza virus-infected P58^IPK−/−^ mice at 1 day post infection. Gene Set Enrichment Analysis (GSEA) analysis was performed on a sample set derived from P58^IPK−/−^ and wild-type mice infected with the PR8 strain of influenza virus in triplicate at 1 day post infection. All infected samples were compared to genotype-matched mock-infected samples via microarrays analysis. Replicate samples were then *in silico* error-weighted pooled and re-ratioed to compare P58^IPK−/−^ gene expression to wild-type gene expression. Following GSEA analysis using only those genes which were significantly regulated, the top gene ontology categories related to the immune response were selected (see Figure S7 for entire table). An edge is placed between gene ontology categories if they share common genes, and the edge's thickness increases as the number of common genes increases. The log_10_ ratio of P58^IPK^ to wild-type gene regulation is noted for selected gene ontology categories.

We found that GO categories at the top of the day 3 list were characterized by the negative regulation of metabolic processes, whereas those at the top of the day 5 list included a random assortment of biochemical processes (Figure S5 and Figure S6). The top of the GSEA list for day 1 included many processes associated with the immune and inflammatory responses (Figure S7). A subset of GO categories, highlighted in Figure S7, is shown in Figure 4, and these categories are connected by edges, the thickness of which corresponds to the number of overlapping genes in each category. The gene expression data showed a dramatic increase in chemokine expression in P58^IPK−/−^ mice, which likely resulted in an exaggerated inflammatory response [26]. Although some gene expression values were low, even small changes in gene expression can have a drastic effect on biological function [27]. In addition, GSEA indicated that the GO categories were significantly perturbed, even if the genes within the categories were not highly regulated. Together, our gene expression analyses showed that the absence of P58^IPK^ during influenza virus infection caused an increase in inflammatory, immune response, and cell death-related genes at early times post infection, likely prognostic of the eventual increased lung pathology and ultimately death.

### P58^IPK−/−^ mice exhibit increased levels of IL-6 and IFNβ in response to infection

**Figure 5:**
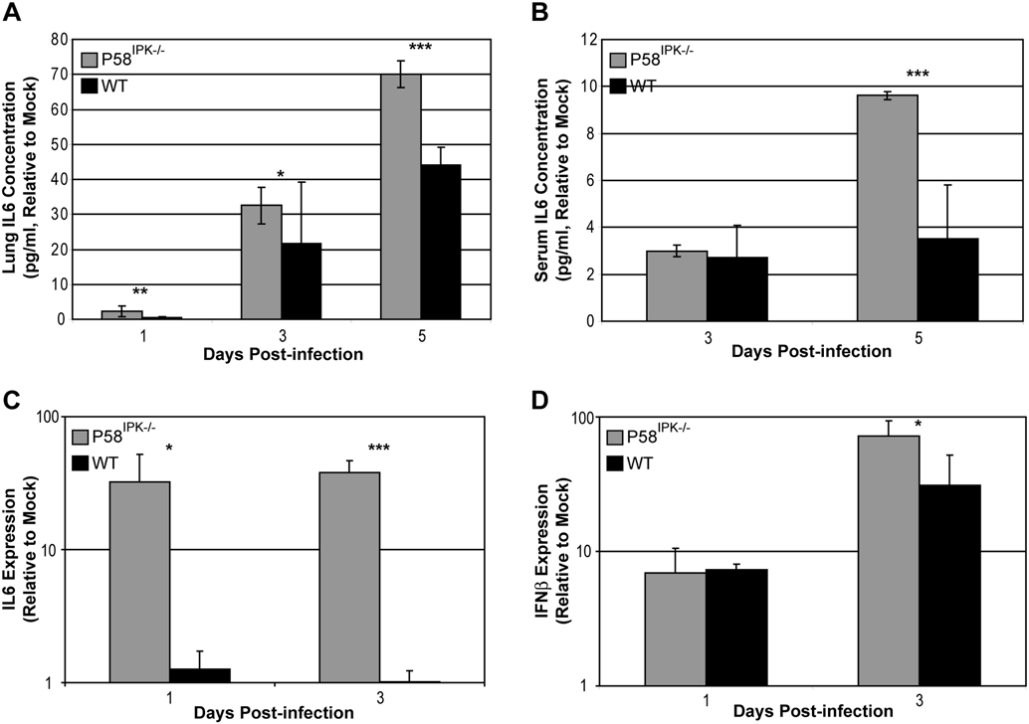
Mice lacking P58^IPK^ display increased levels of IL6 and IFNβ during influenza virus infection. Three P58^IPK−/−^ and wild-type mice were mock infected or infected with 10^3^ (A,B) or 10^4^ (C,D) PFU of the PR8 strain of influenza virus. At 1, 3, or 5 days post infection, blood serum was isolated, azygous and apical lung lobes, or left lung lobes were excised and homogenized. Equivalent concentrations of protein in serum (A) or azygous and apical lung homogenates (B) were subjected to ELISA using an IL6 antibody. Total RNA was isolated from left lung lobes for reverse transcription to generate cDNA. Quantitative RT-PCR was used to determine the amount of mouse IL6 (C) and IFNβ (D) mRNA in each sample. The results represent the mean activity of 3 independent samples (with technical triplicates)±standard deviation. *P* values from a two-tailed *t*-test assuming non-equal variance are indicated (*: *P*<0.05, **: *P*<0.01, ***: *P*<1×10^−4^).

Since genomic profiling indicated an increased inflammatory response in P58^IPK−/−^ mice, we next evaluated whether mice lacking P58^IPK^ exhibited increased cytokine abundance at the protein level. One important cytokine that has been shown to be over-expressed during fatal influenza virus infection is interleukin-6 (IL-6) [28]. Using an enzyme-linked immunosorbent assay (ELISA), we found that IL-6 levels were increased in the lungs of P58^IPK−/−^ mice compared with wild-type animals throughout the course of infection (Figure 5A). Furthermore, we detected increased levels of IL-6 in the serum of infected P58^IPK−/−^ mice at day 5 post infection (Figure 5B). This increase in IL-6 protein in the serum and lungs of infected P58^IPK−/−^ mice was accompanied by a drastic increase in IL-6 mRNA. (Figure 5C). Furthermore, levels of IFNβ mRNA were significantly increased in the lungs of P58^IPK−/−^ mice at day 3 post infection (Figure 5D). IFNβ induction results in the increased production of IL-6 [29], and together, the expression of these two cytokines suggests that a greater IFN and cytokine response contributes to the increased lung pathology and higher mortality rate associated with influenza virus infection of P58^IPK−/−^ mice.

### Pandemic influenza virus infection results in an amplified inflammatory response in P58^IPK−/−^ mice

**Figure 6:**
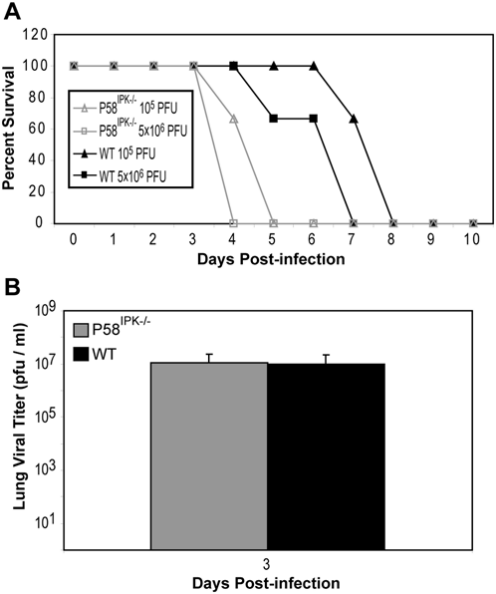
P58^IPK−/−^ mice exhibit increased mortality to pandemic influenza virus infection yet similar levels of virus replication. (A) Three P58^IPK−/−^ and wild-type mice were infected with 10^5^ or 5×10^6^ PFU of the r1918 strain of influenza virus. The number of surviving mice, as determined by having at least 20% of starting weight, at each day is shown. (B) Three P58^IPK−/−^ and wild-type mice infected with 10^4^ PFU of r1918 were sacrificed at 3 days post infection. Levels of infectious virions in diaphragmatic lung homogenates were determined by triplicate plaque assay on MDCK cells. The results represent the mean activity of 3 independent samples±standard deviation.

In addition to using P58^IPK−/−^ mice to determine the role of P58^IPK^ during influenza virus infection, we were also interested in determining whether these mice could provide new insight into the unusually high virulence of the virus responsible for the 1918 influenza pandemic. Infection with 5×10^6^ PFU of r1918 resulted in the death of all P58^IPK−/−^ mice by day 4 post infection and the death of all wild-type animals by day 7 (Figure 6A). This corresponded to an LD_50_ of 10^1.8^ PFU for P58^IPK−/−^ mice and 10^2.5^ PFU for wild-type mice. Similar to PR8, wild-type mice required five times the dose of r1918 to achieve fifty percent mortality, as compared to P58^IPK−/−^ mice. The increased mortality of P58^IPK−/−^ mice was again not due to differences in viral load, since viral titers were nearly identical in P58^IPK−/−^ and wild-type mice (Figure 6B). Microscopic examination revealed lung pathology similar to that reported previously [22],[30].

We then used global gene expression profiling to determine the transcriptional response of P58^IPK−/−^ mice to infection with r1918 and to evaluate similarities and differences in the host response to r1918 and PR8. We began our analysis by comparing lung gene expression profiles of r1918-infected and mock-infected P58^IPK−/−^ mice and selecting for genes that were expressed at a higher level during virus infection. We then determined which genes among this group were expressed at a lower level in r1918-infected wild-type mice. Because these genes were induced in P58^IPK−/−^ mice in response to virus infection, but were expressed at a lower level in virus-infected wild-type mice, the infection-induced expression of these genes appears to be impacted by the presence or absence of P58^IPK^.

**Figure 7:**
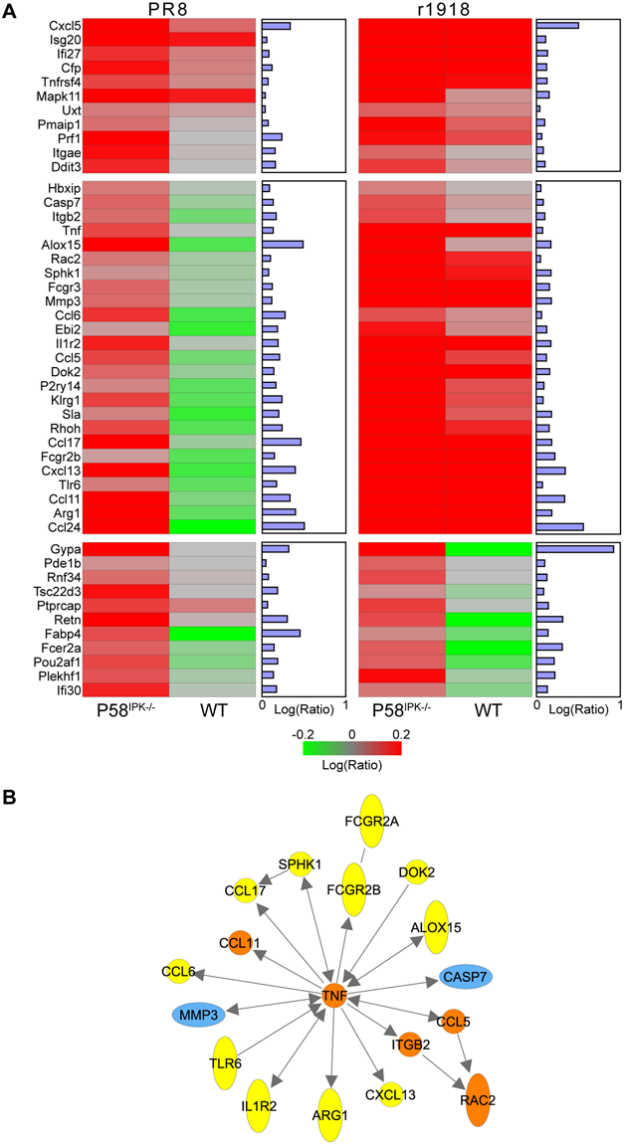
Both PR8 and r1918 infections result in an exaggerated inflammatory response in infected P58^IPK−/−^ mice. (A) Venn diagram analysis was performed on a sample set derived from P58^IPK−/−^ and wild-type (WT) mice infected with the PR8 or r1918 strains of influenza virus in triplicate at 1 day post infection. All infected samples were compared to genotype-matched mock-infected samples via microarrays analysis. Replicate samples were then pooled and error-weighted *in silico*. For each virus, a gene set was isolated which included genes which were up-regulated in P58^IPK−/−^ mice as compared to mock and as compared to wild-type mice. The intersection of the sets for both PR8 and r1918 infection was isolated for IPA. Of the 113 genes in this set, 65 had annotation, and 47 of those had functions related to the inflammatory and cell death responses; log_10_ ratio regulation of these genes in P58^IPK−/−^ and wild-type mice as compared to mock is represented for both viral infections. The bar graph represents the log_10_ ratio of P58^IPK−/−^ to wild-type gene regulation, showing that each gene is more up-regulated in P58^IPK−/−^ mice. (B) IPA network analysis of the middle set of genes in (A) highlights a subset of genes that are up-regulated in wild-type mice during r1918 infection but not PR8 infection. This diagram shows the direct or indirect interactions reported for these cell-death- (blue shading) and inflammatory-response-related (yellow shading) genes. Genes shaded orange fit into both categories.

This same analysis path was then used to analyze the gene expression data generated from the PR8 infections described above, again resulting in a set of infection-induced genes, the expression of which appears to be impacted by the presence or absence of P58^IPK^. Of the annotated genes in this set, 65 were also present in the final set of genes identified from the r1918 virus infection data. The majority of these genes (47 of 65) are associated with inflammatory, immune response, and cell-death pathways and their expression patterns in P58^IPK−/−^ and wild-type mice are shown in Figure 7A. Because these genes were all expressed at a higher level in P58^IPK−/−^ mice, they may therefore be associated with the increased pathology and mortality rate observed in these animals. However, of particular interest are the set of genes present in the middle panel of this Figure, which were induced in P58^IPK−/−^ mice in response to infection with either PR8 or r1918, but which were induced in wild-type mice only in response to the r1918 virus. To evaluate the functional relationships of these genes more closely, we used Ingenuity Pathways Analysis (IPA) to create a network of the set of genes in the middle of Figure 7A, showing direct or indirect interactions reported for these cell-death- and inflammatory-response-related genes (Figure 7B). The network is centered around tumor necrosis factor (Tnf), which is interesting because activation of Tnf-related pathways has been shown to be associated with H5N1 influenza viral infections of macrophages [31]. This pattern of expression suggests that these genes may also contribute to the exceptional virulence of the r1918 virus, but, more importantly, they may contribute to the virus' exceptional virulence in animals lacking P58^IPK^.

## Discussion

In this study, we demonstrate that P58^IPK^ plays a novel role in regulating the antiviral and inflammatory responses to influenza virus infection. Here, we have specifically shown, through detailed high-throughput and biochemical analysis, how P58^IPK^ may protect the host during influenza virus infection. In previous reports, we demonstrated that P58^IPK^ is activated upon infection with influenza virus [32] and that P58^IPK^ binds to and inhibits PKR [7],[33]. We also showed that cells devoid of P58^IPK^ exhibit higher levels PKR activation and eIF2α phosphorylation, yet lower levels of viral mRNA translation during influenza virus infection [8]. Together, these results suggest that P58^IPK^ activation is required for more efficient influenza virus replication *in vitro* and that the virus usurps P58^IPK^ activation to the detriment of the host. However, our current study has expanded and changed our understanding of how the activation of P58^IPK^ impacts viral replication since we have now shown that the absence of P58^IPK^ did not affect levels of viral load *in vivo*, although we did not examine rates of viral replication. Nevertheless, influenza virus infection in P58^IPK−/−^ mice resulted in increased mortality due to a heightened inflammatory response marked by increased lung pathology and apoptosis. Furthermore, at the lowest doses of influenza virus, wild-type mice recovered from infection, suggesting that P58^IPK^ benefits the host during infection.

**Figure 8:**
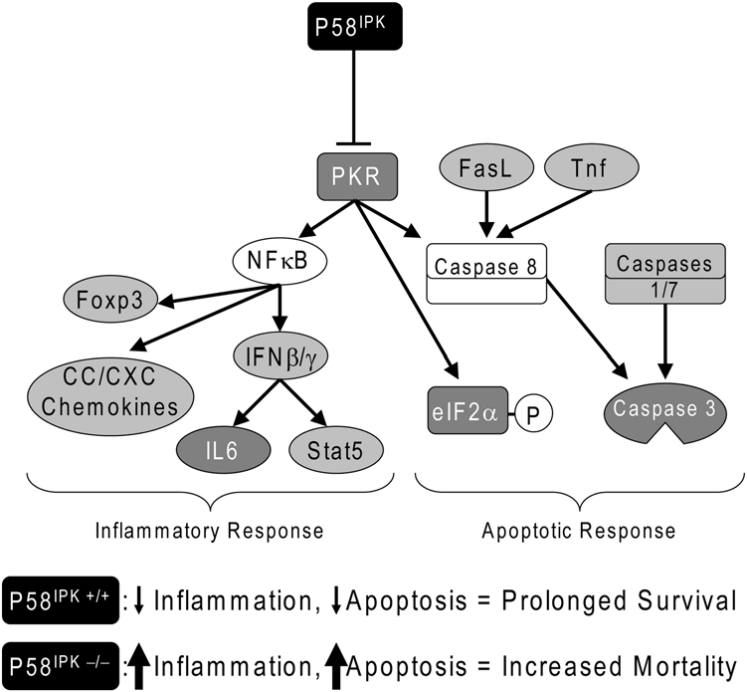
P58^IPK^ functions as an inhibitor of the apoptotic and inflammatory responses during influenza virus infections. During influenza virus infection, P58^IPK^ and PKR are activated. While P58^IPK^-mediated inhibition of PKR results in decreased apoptotic and inflammatory responses, the absence of P58^IPK^ results in the amplification of these responses via increased Caspase, eIF2α, and NF-κB activation. Molecules shaded light grey were up-regulated in infected P58^IPK−/−^ mice at the transcript level while those shaded dark grey were up-regulated at the protein level. In tandem, the enhancement of these responses results in increased mortality in P58^IPK−/−^ mice.

Our findings provide the first direct evidence that P58^IPK^ is a regulator of the host innate immune response repertoire and that activation of P58^IPK^ results in decreased lung pathology and host mortality. A model describing these results is presented in Figure 8, which is divided into two categories: the apoptotic and inflammatory responses. P58^IPK^ functions through each via its inhibition of PKR. P58^IPK^ inhibits the apoptotic response via its inhibition of PKR-mediated caspase activation and eIF2α phosphorylation. P58^IPK^ also inhibits the inflammatory response via its inhibition of PKR-mediated NF-κB activation, which ultimately activates IFN-stimulated genes. Therefore, P58^IPK^ is necessary to counterbalance the activities of PKR, and together, the over-activation of pathways downstream of PKR results in increased host pathology and mortality [20],[28]. While our analysis revealed that neither PERK nor its downstream targets were activated during influenza virus infection, we regard these negative results as inconclusive due to the difficultly in performing these experiments, especially with regard to sample preparation [34]. Since influenza virus hemagglutinin has been shown to cause ER stress [35], we are performing experiments with more sensitive assays to look for markers of ER stress in influenza virus-infected mice.

There is evidence of a connection between PKR and proteins involved in apoptosis, namely FADD and caspase 8 [36],[37], and PKR may engage a death receptor that is upstream of caspase 8 activation and apoptosis. Although a definite connection between PKR and FADD-mediated caspase 8 activation remains to be determined, apoptosis is induced by the over-expression of PKR [38]. Additionally, caspase 8 activation results in caspase 3 activation, resulting in apoptosis [39]. Fas ligand (FasL), Tnf, and caspases 1 and 7, which activate caspases 8 and 3 [40]–[43], were identified in our microarray analysis as being more up-regulated in P58^IPK−/−^ mice during influenza virus infection. We also previously demonstrated that P58^IPK^ is anti-apoptotic, as nude mice injected with cells over-expressing P58^IPK^ undergo malignant transformation [19]. Finally, P58^IPK−/−^ mice, which have basal levels of ER stress [12],[13], exhibit increased caspase 3 activation and apoptosis of β-cells in the pancreas [44]. We have now shown that the stimulus of influenza virus infection in lung tissue contributes to increased apoptosis in the absence of P58^IPK^. This was marked by increased and prolonged eIF2α phosphorylation [21] and increased caspase 3 activation in the lungs of these mice.

With respect to the inflammatory response arm of the model, PKR activates NF-κB, which in turn activates Foxp3, IFNβ, and IFNγ, and a number of chemokines [18],[45],[46]. Each of the downstream NF-κB responses shown in Figure 8, among many others, was up-regulated in P58^IPK−/−^ mice as compared with wild-type mice. Again, this is likely due to uncontrolled activation of PKR in the absence of P58^IPK^. Treatment of bronchial epithelial cells with dsRNA activates both PKR and NF-κB, which results in the secretion of pro-inflammatory interleukins and chemokines [47]. This response could lead to increased lung pathogenesis during a viral infection, which was a striking difference between influenza virus-infected P58^IPK−/−^ and wild-type mice. While only one lung lobe was analyzed for each mouse, and neither step sectioning nor bronchoalveolar lavages were performed, pathogenesis in P58^IPK−/−^ mice was marked by increased macrophage infiltration and hyaline membrane formation. Hyaline membranes obstruct the exchange of oxygen across the alveolar walls and is characteristic of diffuse alveolar damage [48]. It is also a characteristic sign of lethal influenza virus infections in cynomolgus monkeys [49]. In culture, influenza virus infection of macrophages stimulates the release of IL-6 [50], and we observed an increase in IL-6 mRNA and protein levels in the lungs of virus-infected P58^IPK−/−^ mice due to increased macrophage infiltration in the lungs of these animals. Increased levels of IL-6 have also been associated with fatal influenza virus infections [51],[52], which is consistent with the increased mortality observed in P58^IPK−/−^ mice. This is especially significant because it was at day 5 post infection, when IL-6 levels were highest, that mice lacking P58^IPK^ died from infection. IFNβ expression has been shown to be correlated with IL-6 expression during influenza virus infection and severe acute respiratory syndrome (SARS)-coronavirus infection [53],[54], and IFNβ expression was also elevated in influenza virus-infected P58^IPK−/−^ mice.

Antiviral and inflammatory responses are typically necessary to clear the virus and to recover from infection [55]. However, during infection with a virus such as Ebola virus, the marked over-expression of genes associated with the immune and inflammatory response may contribute to disease pathology and death [56],[57]. We have also shown that infection of mice or macaques with H5N1 avian influenza viruses, or the 1918 pandemic virus, results in rapid disease and death, most likely due to an early and dysregulated host inflammatory response, which is associated with severe lung disease and mortality [22],[31],[52],[58],[59]. Our current genomic profiling data suggest a similar phenomenon is occurring in P58^IPK−/−^ mice infected with influenza virus, where the significant expression of immune response-related genes at 1 day post infection was correlated with eventual death of the animal. In contrast, the expression profiles from wild-type mice exhibited increased expression of inflammatory response genes, but not until later times post infection, which was correlated with prolonged survival. Not only did these observations hold for the mouse-adapted PR8 virus, but also for the human 1918 pandemic strain. This is noteworthy, because two strains of influenza virus, each with different species specificity, produced the same disease phenotype in P58^IPK−/−^ mice via the same pathway. Furthermore, we identified a subset of genes, focused largely around Tnf and matrix metallopeptidase 3 (Mmp3), which were up-regulated in wild-type mice only during r1918 infection and not PR8 infection. Although it has been shown that Tnf activation increases the expression of Mmp3 [60], and that matrix metallopeptidases are up-regulated in airway epithelium disease in which macrophages and eosinophils are highly present [61], it has not previously been shown that Mmp3 activation is a function of influenza virus infection. This suggests that even by 1 day post infection, lung cells are undergoing a remodeling process which will ultimately lead to pathogenesis and mortality, by a potentially novel pathway, during pandemic influenza virus infection.

Together, our results show that P58^IPK^ plays an important role in regulating the innate immune response, as its presence results in a more controlled response to influenza virus infection. While P58^IPK^ benefits the host through reduced lung pathology and prolonged survival, it also provides the virus with additional time to replicate and spread among other hosts. The results of our study are therefore somewhat different than those observed in plant virus infection models, where plants lacking P58^IPK^ also exhibited increased host death. Since plants lacking P58^IPK^ exhibited lower levels of viral replication, it was concluded that P58^IPK^ is required for virulence and acts a susceptibility factor [9]. However, unlike animals, plants do not invoke cell death as part of their immune response to virus infection. Since we have shown that in wild-type mice P58^IPK^ functions to modulate the innate immune, inflammatory, and cell death responses during viral infection, resulting in decreased pathology and a lower mortality rate, activation of mammalian P58^IPK^ actually functions to reduce the virulence of influenza virus infection. Thus, we have identified P58^IPK^ as a new class of molecule that is activated during virus infection to inhibit the over-activation of inflammatory and cell death responses in order to prolong host survival. Further studies as to how to modulate the P58^IPK^ pathway, and thus the host's antiviral and inflammatory response, may lead to the discovery of novel therapeutics for targeted intervention during a pandemic influenza virus infection.

## Materials and Methods

### P58^IPK−/−^ mice, viruses, and cells

P58^IPK−/−^ mice were 100% C57BL/6 (from a C57BL/6 embryonic stem cell line) and were maintained on the C57BL/6 background by alternate generation backcrossing as previously described [44]. Mice were maintained in a specific pathogen-free barrier facility with standard rodent diet and 12 h alternating light and dark cycles. The PR8 strain of influenza virus was grown in 10-day-old embryonated chicken eggs [62]. The r1918 influenza virus was generated as previously described [22],[30]. Plaque assays were performed using Madin–Darby canine kidney (MDCK) cells grown as monolayers in high glucose Dulbecco's modified Eagle's medium supplemented to contain 10% heat-inactivated fetal calf serum, 2 mM L-glutamine, penicillin G (50 units/ml), and streptomycin sulfate (50 µg/ml).

### Mouse infections

Ten- to twelve-week-old P58^IPK−/−^ or wild-type C57BL/6 mice (Charles River Laboratories) were anesthetized with isoflurane and infected intranasally with 100 µl aliquots of PBS (control) or PBS containing 10^0^ to 10^5^ plaque-forming units (PFU) of the PR8 strain of influenza virus. On days one, three, and five post infection, three mock-infected and three P58^IPK −/−^ or wild-type mice infected with 10^3^ or 10^4^ PFU were sacrificed. Remaining animals were weighed each day for ten days post infection and sacrificed when they lost at least 20% of their starting body weight. Blood and lung tissue was collected from each mouse at the time of sacrifice. All experiments were performed in a specially separated negative-pressure HEPA (high-efficiency particulate air)-filtered biosafety level 2 laboratory. All animals were handled in strict accordance with good animal practice as defined by the relevant national and/or local animal welfare bodies, and all animal work was approved by the University of Washington Institutional Animal Care and Use Committee. Infections of P58^IPK−/−^ and wild-type C57BL/6 mice with r1918 was performed as previously described [22].

### Plaque assay and protein analysis

Diaphragmatic lung lobes from each animal were weighed, homogenized in PBS, and samples were then assayed in triplicate for viral yield by standard plaque assay on MDCK cells. Viral yields were calculated according to the formula: yield_t = x_ = (log_10_(PFU/ml)_t = x_)/(log_10_(PFU/ml)_t = 0_), where *t* is time and *x* is the time post infection.

Azygous and apical lung lobes were homogenized in mammalian extraction buffer (Pierce) supplemented with 1× complete protease inhibitor (Roche), 25 mM β-glycerophosphate, 1 mM Na_3_VO_4_, and 1 mM NaF. Total protein content was determined for clarified lung homogenates using the BCA protein assay kit (Pierce). Homogenates were separated by SDS-PAGE, with the same amount of total protein being loaded into each lane, and then transferred to nitrocellulose paper. Immunoblots were blocked for 1 h in PBS containing 0.5% Tween 20 and 5% nonfat dry milk, washed in PBS containing 0.05% Tween 20, and incubated at 4°C overnight with rabbit-anti-eIF2α[pS^51^] phosphospecific antibody (Biosource International), a rabbit polyclonal antibody recognizing full-length eIF2α (Santa Cruz Biotechnology), or a mouse monoclonal actin antibody (MP Biochemicals) in PBS containing 0.5% Tween 20 and 1% nonfat dry milk. Membranes were washed, incubated for 2 h with horseradish peroxidase-conjugated donkey anti-mouse or anti-rabbit immunoglobulin G (Jackson Immunoresearch), and bound antibodies detected with ECL Western blotting detection reagent (Amersham Biosciences/GE Healthcare). To determine levels of PKR and phosphorylated PKR, 400 µg of total protein from lung homogenates were immunoprecipitated with an antibody recognizing total PKR (Santa Cruz Biotechnology) for 2 h at 4°C followed by an overnight incubation at 4°C with Protein A Sepharose beads (Amersham Biosciences/GE Healthcare). Beads were washed with NETN buffer (20 mM Tris-HCl, pH 8.0, 100 mM NaCl, 1 mM EDTA, and 0.5% NP-40). Western blotting was then performed for total PKR (Santa Cruz Biotechnology) and pT^451^ PKR (Invitrogen) as described by the product analysis sheets.

Blood was also collected at the time of sacrifice and serum was separated by centrifugation. Using equal amounts of total protein in serum or azygous and apical lung lobe homogenates, absolute levels of IL-6 were determined by enzyme-linked immunosorbent assay (ELISA) using the Quantikine assay kit (R&D Systems) as described by their protocol.

### Histopathological, immunohistochemical, and statistical analysis

Cardiac lung lobes were excised, perfused, and fixed in >10 volumes of 10% neutral-buffered formalin (Fisher Scientific) for 48 h. Paraffin embedding, sectioning, and staining were performed by the Experimental Histopathology Shared Resources at the Fred Hutchinson Cancer Research Center (Seattle, WA). 4 micron sections of lung were stained with H&E and PAS by standard methods. Sections were also immunostained for cleaved caspase 3 and macrophage marker F4/80 as described [63]. Influenza virus NP was detected with a rabbit polyclonal antibody (a kind gift from Adolfo García-Sastre) at 1∶8000 and using the protocol described for cleaved caspase 3 without antigen retrieval.

To score lung inflammation and damage, a semi-quantitative scoring system was used; for this, the entire cardiac lobe surface was analyzed with respect to the following parameters: alveolitis, peribronchiolitis, and perivasculitis. Each parameter was graded on a scale of 0–4 with 0 as “absent,” 1 as “slight,” 2 as “mild,” 3 as “moderate,” and 4 as “severe.” The total “lung inflammation scor” for each mouse lung lobe was determined as the sum of the scores for each parameter, the maximum being 12. The average, standard deviation, and *P*-values from a two-tailed *t*-test assuming non-equal variance were determined for each set of triplicate mice for each genotype. Representative micrographs from the most significant doses are presented for both genotypes.

To score immunohistochemical staining, the same method described above was used to score the entire cardiac lobe surface, except that a scoring system of 0–3 was used with 0 as “none,” 1 as “infrequent,” 2 as “common,” and 3 as “widespread.”

### Quantitative RT-PCR

Following animal sacrifice, the left lung lobe was homogenized in Solution D (4 M guanidinium thiocyanate, 25 mM sodium citrate, 0.5% sarcosyl, 0.1 M β-mercaptoethanol), and total RNA was isolated using RNAeasy (Qiagen). The quantity of total RNA was determined by spectrophotometry using a NanoDrop ND-1000 Fluorospectrometer. Contaminating DNA was removed by treating samples with RNAase-*free* DNase and removal reagents (Ambion). Reverse transcription was performed using TaqMan reverse transcription reagents (Applied Biosystems). RT-PCR was performed as previously described [64]. Each target was run in quadruplicate, with 100 ng of sample in reaction volumes of TaqMan 2× PCR Universal Master Mix (Applied Biosystems). Genome copy numbers were normalized to β-actin values determined in parallel using Taqman gene expression assay endogenous control primer-probe sets (Applied Biosystems). Quantification of each gene, relative to the calibrator, was calculated by the instrument, using the equation 2^ΔCT(infected)−ΔCT(mock)^ within the Applied Biosystems Sequence Detection Software version 1.3. The minor groove binding probe and primer sets for each gene were part of an Applied Biosystems assay set as follows: Mouse IL6: Mm00446190_m1; Mouse IFNβ: Mm00439546_s1.

### Expression microarray analysis and bioinformatics

Amplification of mRNA was performed as described previously using equal masses of total RNA isolated from left lung lobes of infected mice [57]. An equal-mass pool of mRNA isolated from the lungs of five individual mock-infected mice was prepared as a reference sample. Microarray slide hybridization was performed using mouse oligonucleotide genome CGH arrays (G4426B; Agilent Technologies). For each infection group, expression oligonucleotide array analysis was performed using RNA isolated from lung tissue from three individual animals. The data presented are the error-weighted average changes in expression calculated from four technical replicate arrays performed on three individual mice. All data were entered into a custom-designed relational database and subsequently uploaded into Rosetta Resolver System 7.1 (Rosetta Biosoftware), Spotfire Decision Site 9.1 (Spotfire/Tibco), or Ingenuity Pathways Analysis (Ingenuity Systems, Inc.). Primary microarray data are available at http://viromics.washington.edu.

Gene set enrichment analysis was performed as described [24] using only those genes which were significantly regulated and not taking into consideration the first instance of the maximum enrichment score. *P* values were calculated using the dynamic programming method as described [65].

## Supporting Information

Figure S1 Pathology increases at a greater rate in mice lacking P58^IPK^. P58^IPK−/−^ and wild-type mice were mock infected or infected with 10^3^ PFU of the PR8 strain of influenza virus. At 1, 3, and 5 days post infection, cardiac lung lobes were excised and fixed in 10% neutral-buffered formalin. Lobes were paraffin embedded, sectioned, and stained for hematoxalin and eosin. Bar = 50 µm.
(7.46 MB TIF)

Figure S2 Macrophage infiltration occurs at a greater rate in mice lacking P58^IPK^. P58^IPK−/−^ and wild-type mice were mock infected or infected with 10^3^ PFU of the PR8 strain of influenza virus. At 1, 3, and 5 days post infection, cardiac lung lobes were excised and fixed in 10% neutral-buffered formalin. Lobes were paraffin embedded, sectioned, and stained for the macrophage marker, F4/80. Bar = 50 µm.
(6.98 MB TIF)

Figure S3 Mice lacking P58^IPK^ do not exhibit increased levels of viral protein throughout infection. P58^IPK−/−^ and wild-type mice were mock infected or infected with 10^3^ PFU of the PR8 strain of influenza virus. At 1, 3, and 5 days post infection, cardiac lung lobes were excised and fixed in 10% neutral-buffered formalin. Lobes were paraffin embedded, sectioned, and stained for influenza virus NP. Bar = 50 µm.
(6.35 MB TIF)

Figure S4 Caspase 3 is activated in mice lacking P58^IPK^ during influenza virus infection. P58^IPK−/−^ and wild-type mice were mock infected or infected with 10^3^ PFU of the PR8 strain of influenza virus. At 1, 3, and 5 days post infection, cardiac lung lobes were excised and fixed in 10% neutral-buffered formalin. Lobes were paraffin embedded, sectioned, and stained for cleaved caspase 3. Bar = 50 µm.
(6.59 MB TIF)

Figure S5 Day 3 Gene Set Enrichment Analysis (GSEA) table. Gene ontology categories are shown if there are 3 or more genes in the category. Categories highlighted in grey represent those discussed in the text.
(1.16 MB TIF)

Figure S6 Day 5 Gene Set Enrichment Analysis (GSEA) table. Gene ontology categories are shown if there are 3 or more genes in the category.
(1.22 MB TIF)

Figure S7 Day 1 Gene Set Enrichment Analysis (GSEA) table. GSEA table from the analysis described in Figure 4. Gene ontology categories are shown if there are 3 or more genes in the category. Categories highlighted in grey are used for the analysis in Figure 4.
(1.04 MB TIF)
