# Supplementary figures and images for: P58IPK: A Novel “CIHD” Member of the Host Innate Defense Response against Pathogenic Virus Infection
Source: PLoS Pathog. 2009 May 22;5(5):e1000438. doi: 10.1371/journal.ppat.1000438 (PMC2677460; doi:10.1371/journal.ppat.1000438)

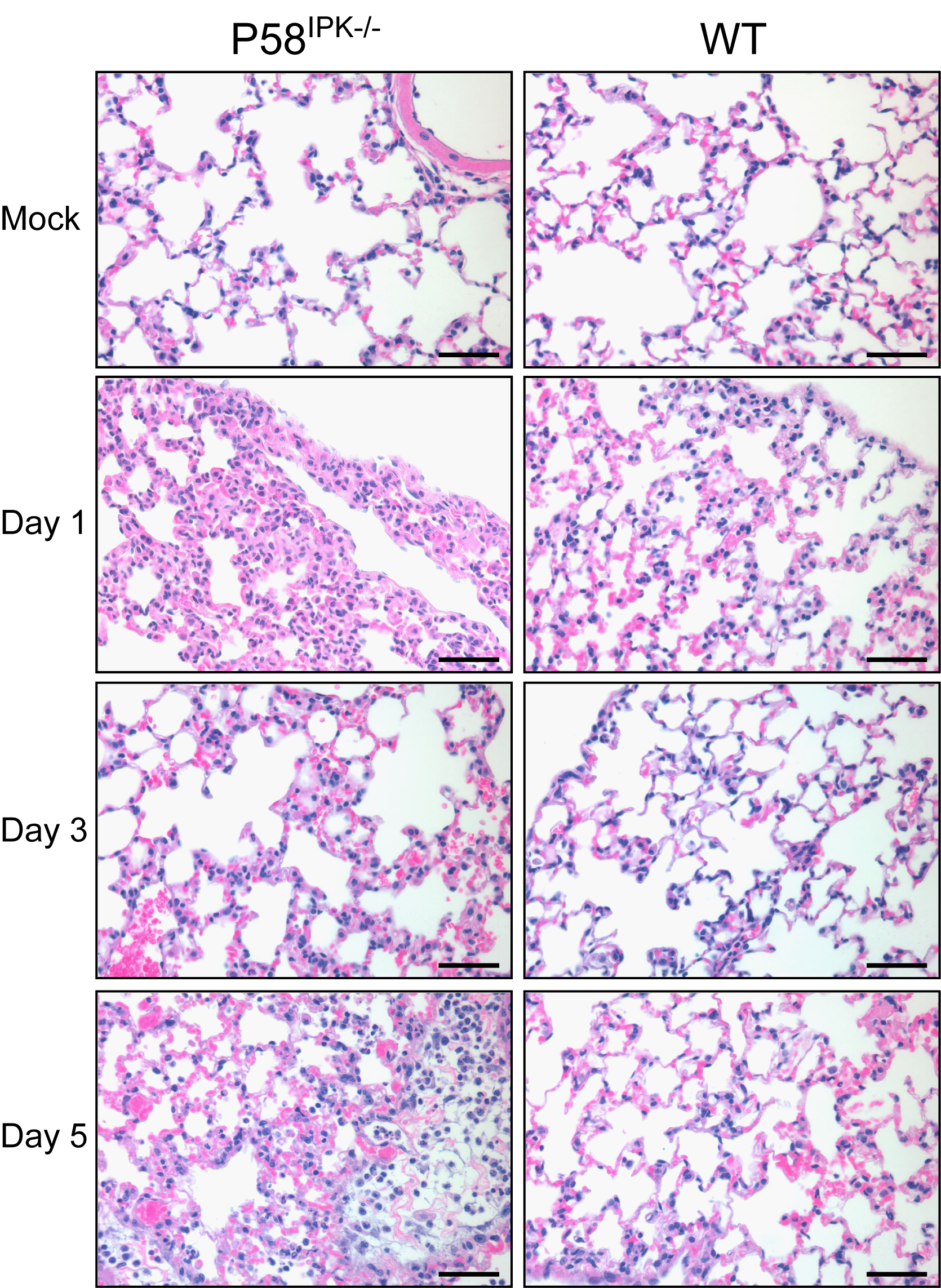

Supplement: Figure S1 — Pathology increases at a greater rate in mice lacking P58IPK. P58IPK−/− and wild-type mice were mock infected or infected with 103 PFU of the PR8 strain of influenza virus. At 1, 3, and 5 days post infection, cardiac lung lobes were excised and fixed in 10% neutral-buffered formalin. Lobes were paraffin embedded, sectioned, and stained for hematoxalin and eosin. Bar = 50 µm. (7.46 MB TIF) [file ppat.1000438.s001.tif]

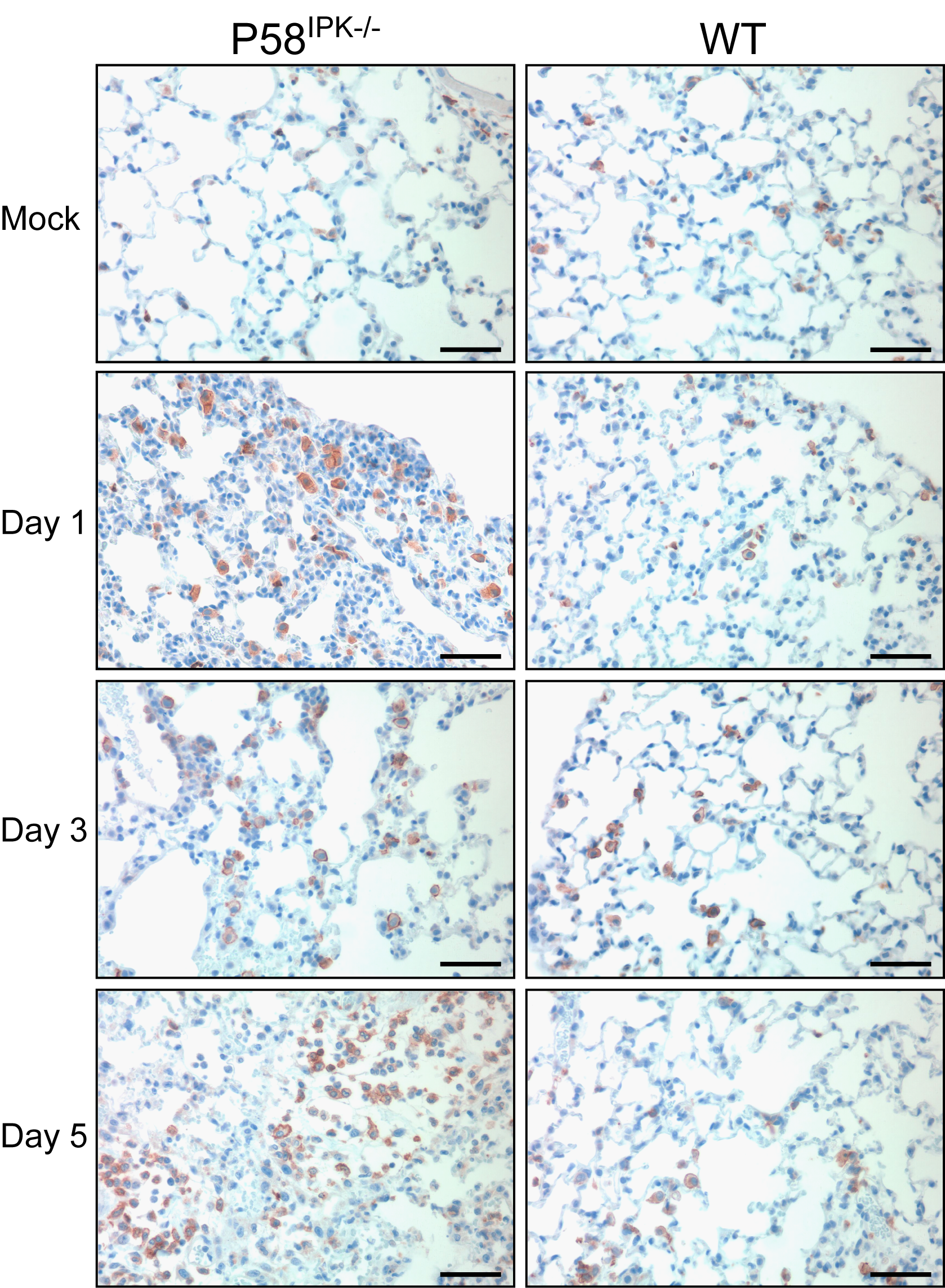

Supplement: Figure S2 — Macrophage infiltration occurs at a greater rate in mice lacking P58IPK. P58IPK−/− and wild-type mice were mock infected or infected with 103 PFU of the PR8 strain of influenza virus. At 1, 3, and 5 days post infection, cardiac lung lobes were excised and fixed in 10% neutral-buffered formalin. Lobes were paraffin embedded, sectioned, and stained for the macrophage marker, F4/80. Bar = 50 µm. (6.98 MB TIF) [file ppat.1000438.s002.tif]

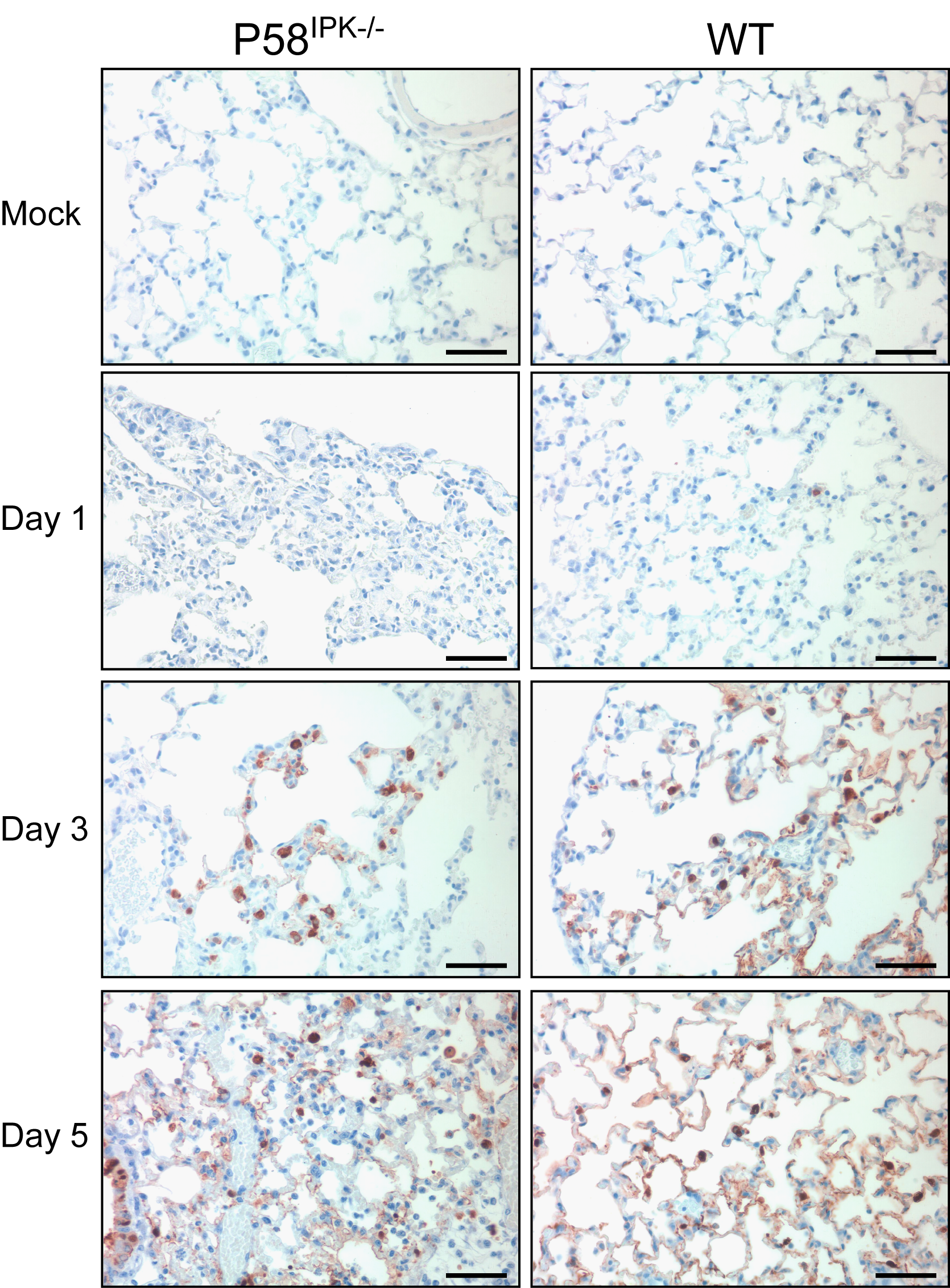

Supplement: Figure S3 — Mice lacking P58IPK do not exhibit increased levels of viral protein throughout infection. P58IPK−/− and wild-type mice were mock infected or infected with 103 PFU of the PR8 strain of influenza virus. At 1, 3, and 5 days post infection, cardiac lung lobes were excised and fixed in 10% neutral-buffered formalin. Lobes were paraffin embedded, sectioned, and stained for influenza virus NP. Bar = 50 µm. (6.35 MB TIF) [file ppat.1000438.s003.tif]

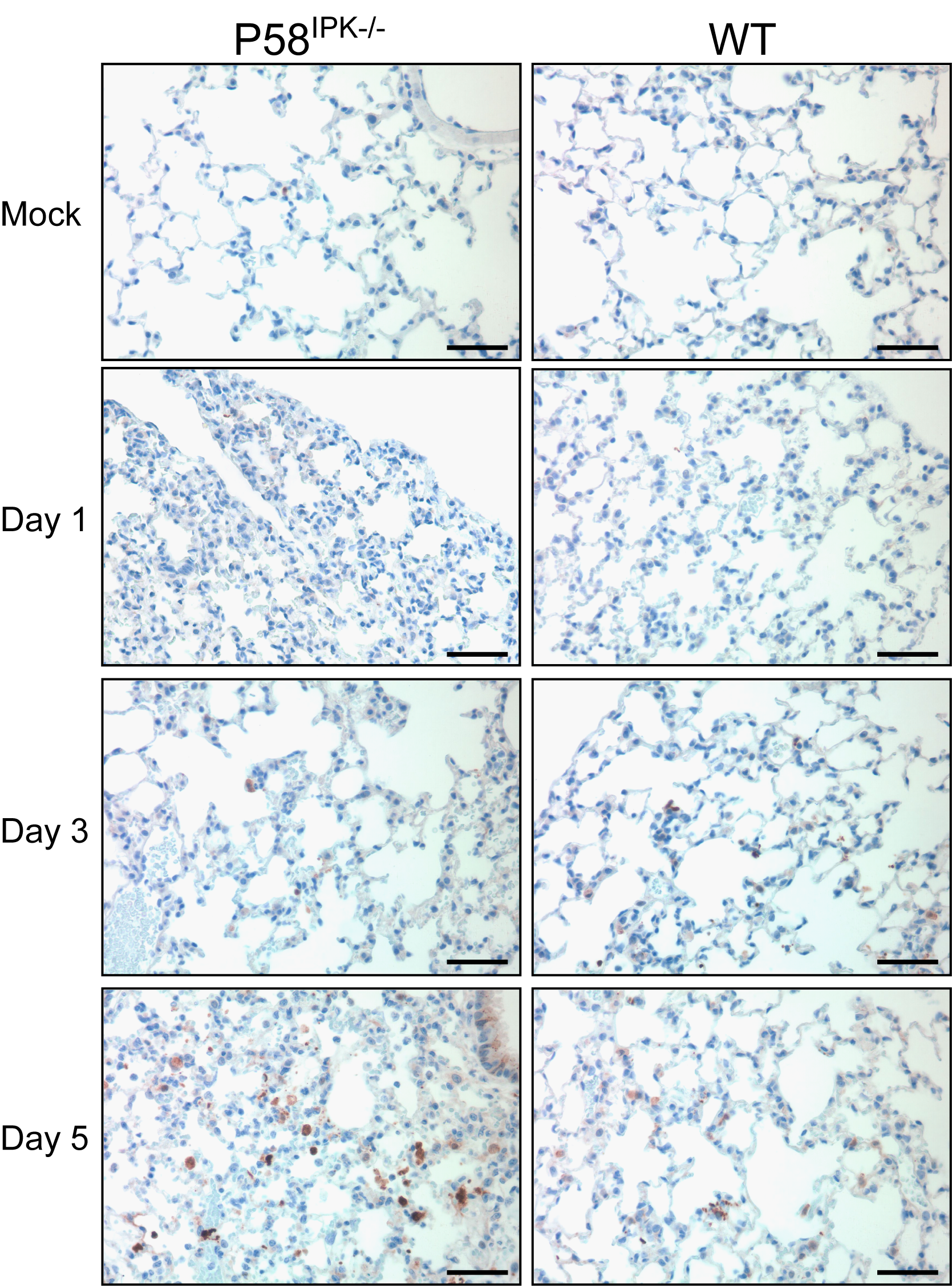

Supplement: Figure S4 — Caspase 3 is activated in mice lacking P58IPK during influenza virus infection. P58IPK−/− and wild-type mice were mock infected or infected with 103 PFU of the PR8 strain of influenza virus. At 1, 3, and 5 days post infection, cardiac lung lobes were excised and fixed in 10% neutral-buffered formalin. Lobes were paraffin embedded, sectioned, and stained for cleaved caspase 3. Bar = 50 µm. (6.59 MB TIF) [file ppat.1000438.s004.tif]

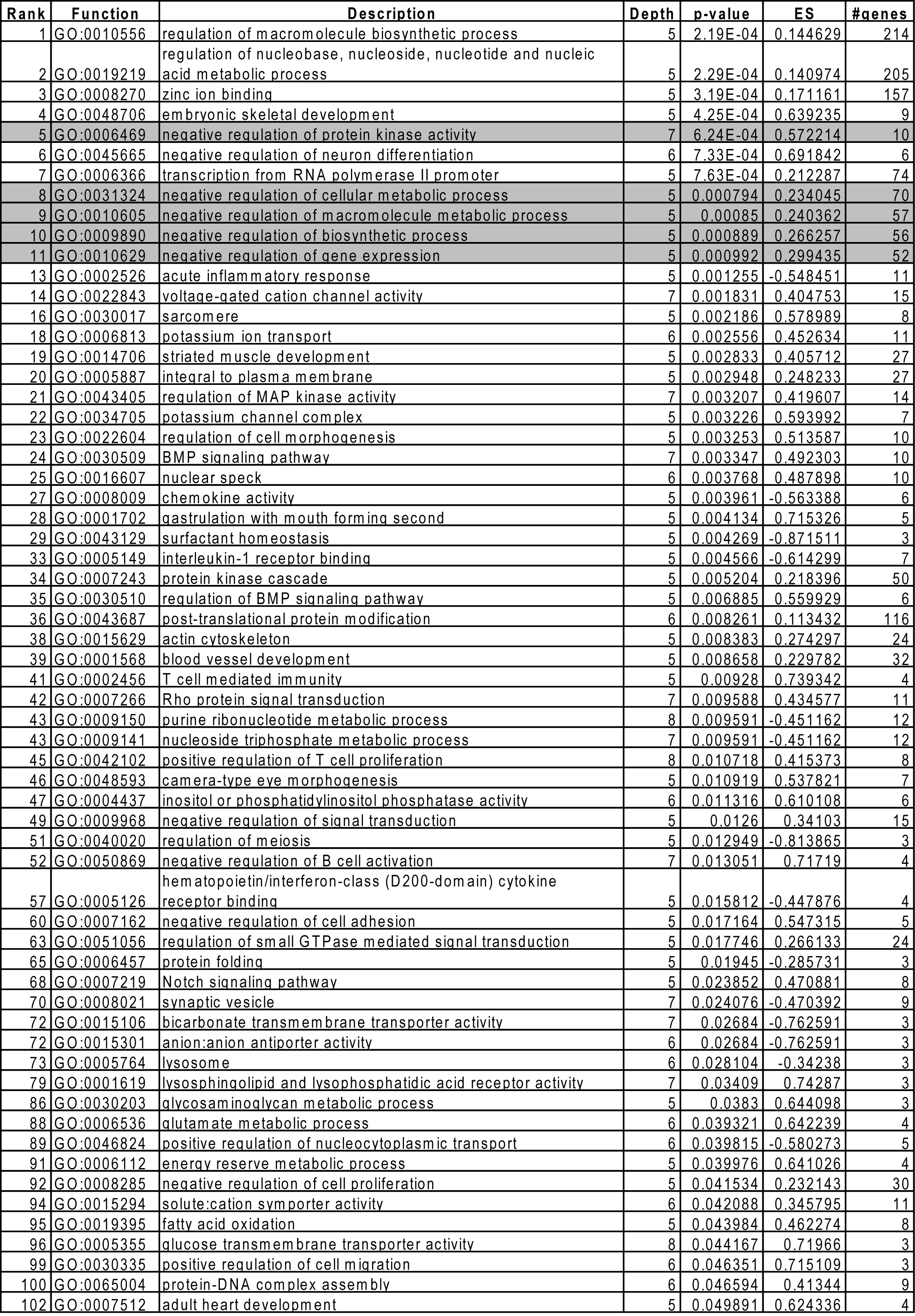

Supplement: Figure S5 — Day 3 Gene Set Enrichment Analysis (GSEA) table. Gene ontology categories are shown if there are 3 or more genes in the category. Categories highlighted in grey represent those discussed in the text. (1.16 MB TIF) [file ppat.1000438.s005.tif]

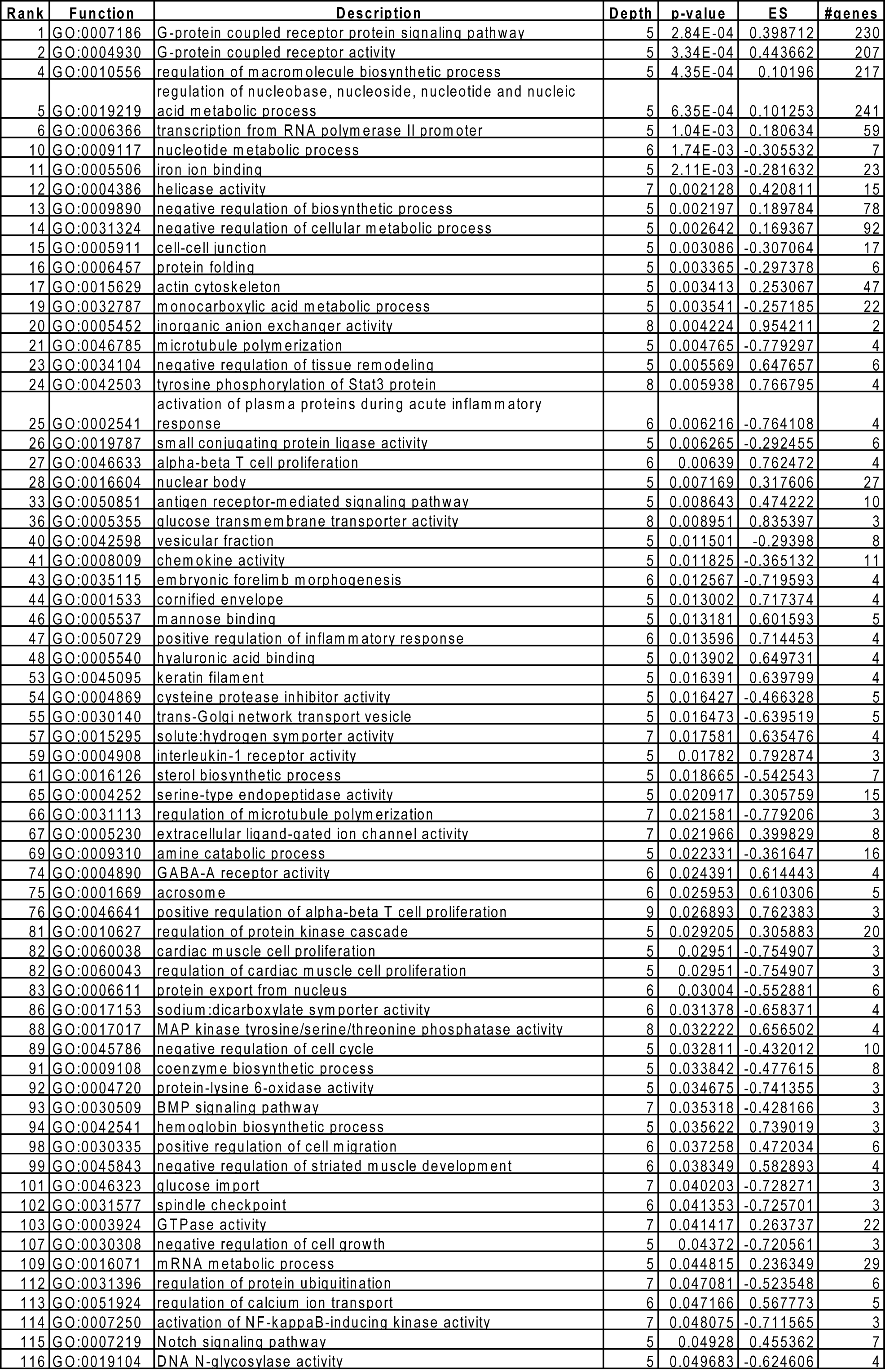

Supplement: Figure S6 — Day 5 Gene Set Enrichment Analysis (GSEA) table. Gene ontology categories are shown if there are 3 or more genes in the category. (1.22 MB TIF) [file ppat.1000438.s006.tif]

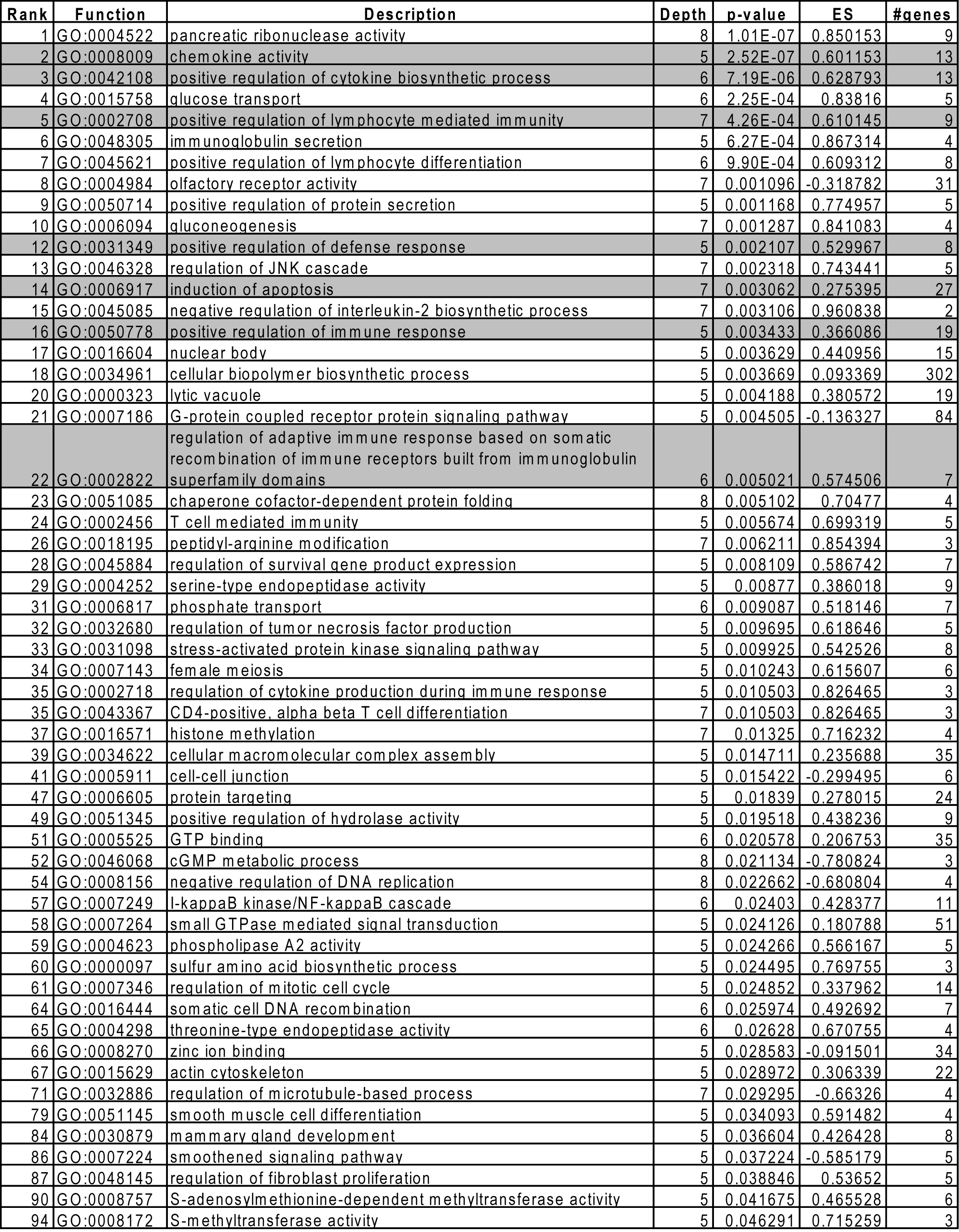

Supplement: Figure S7 — Day 1 Gene Set Enrichment Analysis (GSEA) table. GSEA table from the analysis described in Figure 4. Gene ontology categories are shown if there are 3 or more genes in the category. Categories highlighted in grey are used for the analysis in Figure 4. (1.04 MB TIF) [file ppat.1000438.s007.tif]
